# Defining the Role of Attention in Hierarchical Auditory Processing

**DOI:** 10.3390/audiolres11010012

**Published:** 2021-03-13

**Authors:** Caitlin N. Price, Deborah Moncrieff

**Affiliations:** 1 Institute for Intelligent Systems, University of Memphis, Memphis, TN 38152, USA; dmncreff@memphis.edu; 2 School of Communication Sciences & Disorders, University of Memphis, Memphis, TN 38152, USA

**Keywords:** speech perception in noise, central auditory deficits, theories of attention, electrophysiology

## Abstract

Communication in noise is a complex process requiring efficient neural encoding throughout the entire auditory pathway as well as contributions from higher-order cognitive processes (i.e., attention) to extract speech cues for perception. Thus, identifying effective clinical interventions for individuals with speech-in-noise deficits relies on the disentanglement of bottom-up (sensory) and top-down (cognitive) factors to appropriately determine the area of deficit; yet, how attention may interact with early encoding of sensory inputs remains unclear. For decades, attentional theorists have attempted to address this question with cleverly designed behavioral studies, but the neural processes and interactions underlying attention’s role in speech perception remain unresolved. While anatomical and electrophysiological studies have investigated the neurological structures contributing to attentional processes and revealed relevant brain–behavior relationships, recent electrophysiological techniques (i.e., simultaneous recording of brainstem and cortical responses) may provide novel insight regarding the relationship between early sensory processing and top-down attentional influences. In this article, we review relevant theories that guide our present understanding of attentional processes, discuss current electrophysiological evidence of attentional involvement in auditory processing across subcortical and cortical levels, and propose areas for future study that will inform the development of more targeted and effective clinical interventions for individuals with speech-in-noise deficits.

## 1. Introduction

Attentional selection of relevant inputs is a phenomenon that has been extensively studied in the auditory, somatosensory, and visual sensory modalities. In the auditory domain, attentional selection applies in everyday communicative interactions as the listener must attend to signals of interest while ignoring, or suppressing, competing signals present in the soundscape. Difficulties extracting important speech information from background noise have been linked to a variety of communication disorders in individuals of all ages for several decades. In children, these difficulties may impair a child’s ability to access the phonological structure of language [1,2] and contribute to developmental learning and reading difficulties [3]. In adults with [4,5] and without hearing impairment [6,7], difficulties hearing and communicating in noise may underlie greater social isolation [8], depression [9], poorer quality of life [10], and accelerated cognitive decline [11,12].

Both bottom-up perceptual and top-down cognitive processes contribute to a listener’s ability to extract a salient auditory signal from a competing stream of background information. Uni- and bi-directional components of the auditory brain that contribute to adequate processing of speech in background noise mature at different rates across development and are subject to a variety of acquired factors across adulthood and aging. Moore [13] noted that the ascending pathway matures early in most children with normal hearing but some children develop auditory perceptual skills as late as 10–12 years of age due to slower maturation of top-down mechanisms [13,14].

**Figure 1:**
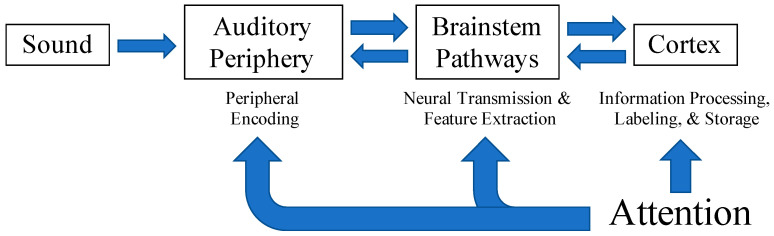
Attention is thought to influence the initial encoding and subsequent processing of auditory inputs across all levels of the auditory system. Adapted from Humes, 1996 with permission from the American Academy of Audiology.

Both Moore [15] and Humes [16] attribute difficulties in auditory perception, particularly in complex listening situations, to underlying pathologies within various levels of the auditory system (depicted in Figure 1). Humes [16] describes these as the peripheral, central-auditory, and cognitive hypotheses. The peripheral hypothesis suggests that changes in the peripheral auditory system result in poorer peripheral encoding of acoustic signals degrading the quality of speech inputs to higher processing centers. The central-auditory hypothesis posits that structural or functional changes within the auditory pathway from the brainstem to auditory cortex result in altered neural transmission and feature extraction vital for the optimization of cues (i.e., temporal, spectral, spatial) to improve speech understanding in complex listening environments. Lastly, the cognitive hypothesis implies that a general cognitive deficit influences the efficiency of information processing, labeling, and storage within memory [16]. A breakdown in processing at any level of the system could lead to errors in perceptual understanding.

As speech-in-noise (SIN) deficits are present even in normal hearing listeners [6,7], these difficulties cannot be attributed to audibility within the peripheral system alone. The underlying issue likely involves a central-auditory or cognitive deficit or an interaction between the two. When the efficiency and synchrony of bottom-up, perceptual processing of auditory stimuli is disrupted, listening and learning difficulties are exacerbated due to an impaired ability to extract necessary acoustic features for comprehension. For instance, school-aged children with listening and learning difficulties demonstrate less precise and robust early sensory encoding of speech signals at the level of the auditory brainstem [17,18,19,20] as well as weaker cortical processing [17] despite normal peripheral hearing sensitivity. Likewise, young adults with normal hearing who perform poorly on SIN tasks demonstrate less robust brainstem [21,22] and cortical responses in noise [22,23], and older adults who are poor SIN performers demonstrate weaker pitch encoding within the auditory brainstem than audiometrically and age-matched peers [24]. These findings suggest that central auditory deficits may contribute to poorer speech-in-noise comprehension across the lifespan.

At the same time, global cognitive processes and functions influence how auditory information is processed and perceived by a listener. Factors such as processing speed [25,26], working and episodic memory [26,27,28], inhibitory control [26,28,29,30], and linguistic knowledge [25,30] affect perceptual performance. While some of these abilities are altered due to age, hearing loss, or auditory processing disorders, other cognitive factors attempt to compensate for associated declines in these areas [25,29]. For instance, age results in reduced processing speed which contributes to poorer temporal processing abilities and ultimately impaired speech perception [25,31]. When linguistic context is added to temporally degraded speech, older adults capitalize on prior linguistic knowledge to improve performance and overcome some of the challenges introduced by typical aging [25]. This suggests that cognitive factors can be both beneficial and detrimental to speech understanding. From prior work, it is evident that cognitive processes work together and interact to form perceptual experiences. The complex interactions between higher-order functions likely contribute to the high degree of variability observed in perceptual abilities across individuals.

Attention is a cognitive process that bridges bottom-up sensory input with identification and perception and contributes to speech understanding in challenging listening conditions. Attention serves to alert and orient us to when and where stimuli of interest occur within our environment [32]. It also allows us to monitor ongoing events or inputs and focus on what is most important while suppressing irrelevant, competing inputs [32]. Each of these functions assists in the prioritization and selection of relevant sensory inputs which is vital to effective communication in noise. Attention is thought to influence the processing of auditory signals from periphery to cortex (see Figure 1) [33,34,35,36,37] via top-down, corticofugal projections from cortical areas to nuclei within the brainstem and to peripheral structures [38,39,40,41,42]. Top-down attentional modulation of early sensory processing via short-term plasticity throughout the ascending pathway and into the auditory cortex may enhance neural representations of auditory signals throughout the auditory pathway [39,43,44,45] and aid behavioral responses and speech comprehension. Alternatively, impairments in attention may be mistaken as peripheral or central auditory processing deficits in certain clinical populations including children with language and learning difficulties [13,15] and older adults [46], making it difficult for clinicians to render appropriate diagnoses and treatments. These studies demonstrate the interconnectedness of peripheral and central auditory processes and higher-order cognitive influences. Further investigation of attentional effects on speech processing throughout the auditory system may provide additional insight into how cognitive processes interact with early auditory processing and contribute to speech understanding.

Such studies would also elucidate at which stages of processing attentional modulations occur. Electrophysiologic studies provide unique insight into the underlying mechanisms of auditory processing across all levels of the auditory neuroaxis. For years, scalp-recorded electrophysiologic measures have been used as a non-invasive method to infer the underlying neural mechanisms contributing to behavior. Electrophysiologic recordings reflect the temporal and spatial summation of neural activity contributing to the processing of sensory inputs and cognitive processes with remarkable temporal resolution [47]. These responses provide a glimpse of the complex interplay between the initial encoding of inputs, neural transmission, and higher order cognitive processes that influence the processing and integration of auditory signals. Importantly, because of their fine temporal resolution and presence throughout the auditory processing hierarchy, electrophysiologic measures are ideal for investigating attentional effects on auditory processing of speech.

Over the course of time, theorists have proposed numerous hypotheses regarding how and when attention plays a role in sensory processing with these attentional effects ultimately affecting perception and behavioral responses. As most of these theories were based on behavioral studies, the application of theoretical suppositions to electrophysiologic studies enables the establishment of brain–behavior relationships and the mapping of selective attention to neuroanatomical structures. This article aims to bridge these literatures by (1) establishing a framework of selective attention by reviewing relevant proposed theories, (2) evaluating electrophysiologic evidence of selective attention throughout the auditory neuroaxis focusing on the controversial attentional effects on early processing within the brainstem, and (3) identifying areas for future research to clarify the potential role of attention in early auditory processing and the clinical implications of such studies.

## 2. Attentional Theories

Selective attention is necessary given the limited amount of information that humans can process at one time. If multiple inputs are presented simultaneously, individuals are only able to consciously detect a few items. Thus, attentional selection reflects an individual’s prioritization of relevant information to account for limitations of the processing system [48,49,50]. The initial study of attentional selection aimed to better understand observed limits in processing capacity. Following these early studies, various attentional theories developed to accommodate emerging contradictory evidence to previously established theories (summarized in Table 1). With each new adaptation, researchers have attempted to encompass the complexities of the interactions between sensory systems and higher order cognitive processes and address how sensory input is selected for further processing and realized as a conscious percept. Overall, the recurrent issue of debate amongst the theories is how and when attentional selection occurs in sensory processing.

### 2.1. Early vs. Late Filter Theories

Early researchers of auditory attention likened sensory processing to communication transmission systems of the time. Early models suggested a limited amount of information could be transmitted along a given channel at one time from the sender to the receiver, yet theorists disagreed about when selective filtering of inputs occurred. Some theorists believed that selective filtering happened early in processing to prevent overloading the channel with excessive inputs [51,52] while others felt filtering occurred later in processing after semantic categorization and classification [53,54].

Proposing an early filter model, Broadbent [51] hypothesized that all sensory inputs are processed in a parallel manner up to a selective filter. The selective filter restricts the amount of information that reaches the limited capacity channel using physical characteristics of the acoustic signal (i.e., frequency, intensity, timing). At this point, processing becomes serial and is considered to be post-categorical, or semantically identified or categorized [55]. Therefore, this model presumes that any information that did not pass through the filter is not semantically processed. Broadbent described the filtering mechanism as “all or nothing” so that the inputs that were filtered out early in processing were not further analyzed. However, a dichotic listening study conducted by Cherry [56] and shadowing studies conducted by Moray [57] challenged this supposition. These studies found that inputs presented to the unattended ear are processed semantically to some degree, particularly if the inputs are subjectively important to the listener. In these studies, listeners were able to identify changes in the speech stream in the unattended ear not related to physical characteristics (i.e., reversal of speech) [56]. Listeners also detected instructions related to shadowing a presented message (i.e., stop shadowing, change attended ear) in the unattended ear more often when the instructions were preceded by their own name [57].

Based on the findings of Cherry and Moray, Treisman [52] expanded Broadbent’s initial model to develop the attenuated filter model which suggests that information presented to the unattended ear is processed to some degree but this information is attenuated relative to the ear to which attention is directed. She provided additional support for semantic analysis of inputs presented to the unattended ear. While Moray’s study focused on words “important” to the participants such as their names, Treisman evaluated the influence of context in running speech. The results revealed that participants’ responses were affected when more context was available within the sentence. When the speech streams were switched between the ears, more shifts in attention, as observed in shadowing errors, were noted in conditions in which greater contextual cues were present in the running speech. Treisman demonstrated that both “important” words and contextual cues resulted in shifts of attention. Furthermore, participants were often not conscious of the shadowing errors made or shifts in their attention to the message in the unattended ear. Overall, these findings suggested that unattended messages are not completely blocked and not processed but rather attenuated.

Deutsch and Deutsch [53] disagreed that the filtering of inputs occurs early in processing and proposed a late filter model. They suggested that all auditory inputs are fully processed with filtering (e.g., attentional selection) occurring after the analysis of physical characteristics and classification into categories. Selective filtering occurs just prior to the behavioral response according to this model. By identifying the most important signal at any given time, this system ensures appropriate response selection for only the most relevant input. The late filtering model also allows for other more important signals, like an alarm, to reach an individual’s awareness even when attention is focused elsewhere. This model implies that the greatest constraint within the system results from limitations in our ability to multitask (i.e., a restricted number of responses can be elicited at a given point in time) and store information in memory for later retrieval and response. On the other hand, early filter models posit that greater limitations are posed by early perceptual processes based on the physical characteristics of the sensory input [58]. For instance, if a listener were engaged in a conversation with a spouse but their children were also present in the room and talking, early filter theories suggest that the listener would only fully process the spouse’s conversation stream based on the frequency, intensity, and temporal characteristics of the spouse’s voice. Late filter theories imply that both the spouse’s and children’s speech streams would be fully processed, but the listener would be limited in his/her response. In this case, the listener can only respond to either the spouse or the children by providing a verbal response or storing the information in memory for later use, but both inputs cannot be responded to or stored in memory simultaneously.

**Table 1:** Description of attentional theories.

Theory Type	Attentional Theory	Description	References
Filter	Early filter theory	All or nothing filtering mechanism Filtering occurs prior to perceptual analysis	Broadbent [51,55]
	Attenuated filter model	Processing of unattended stimuli attenuated relative to attended inputs	Treisman [52,58] Treisman & Geffen [59]
	Late filter theory	All inputs processed fully Filtering occurs prior to response selection	Deutsch & Deutsch [53]
Limited capacity	Limited capacity theory	All inputs fully processed when spare capacity available Attentional selection occurs when task demands exceed capacity	Kahneman [54]
	Framework for Understanding Effortful Listening (FUEL)	Adapts Kahneman’s model to auditory perception providing more direct implications for listening adverse conditions and hearing loss	Pichora-Fuller et al. [60]
	Load theory	System comprised of 2 mechanisms: passive perceptual selection & active cognitive control Attentional selection only occurs under high perceptual load; otherwise, all inputs fully processed	Lavie [61] Lavie et al. [62]

### 2.2. Limited Capacity Theories

Taking a slightly different approach, other theorists focus on the limited capacity of the system rather than the filtering mechanism. Like many early theories of attention, limited capacity theories share similarities to previously proposed theories yet add unique perspective by attempting to address more complex scenarios, particularly by incorporating interactions between perceptual and higher-order cognitive processes. Whereas filter theories propose serial processing mechanisms for attention, capacity theories describe how bottom-up and top-down mechanisms interact to influence attentional selection and behavior.

Kahneman [54] proposed a limited capacity theory which assumes that there are limits in capacity to perform tasks requiring mental processes and the amount of attention that can be actively utilized at a given time is limited. Similar to late filter theorists (i.e., Deutsch and Deutsch), Kahneman [54] explained that all inputs are fully processed as long as spare capacity remains available. However, because the capacity of the system is limited, the amount of spare capacity for processing decreases as attention is directed, or selected, to complete a primary task. Therefore, behavioral performance suffers when the demands of the task exceed the available capacity of attention. This model allows for attention to be divided between two or more tasks while eliciting multiple responses whereas late filter theories suggest that only one response may be selected at a given time.

More recently, Pichora-Fuller et al. [60] adapted Kahneman’s limited capacity model to auditory perception creating the Framework for Understanding Effortful Listening (FUEL). This adaptation describes specific implications for listening in challenging environments and for individuals with hearing loss. Pichora-Fuller et al. [60] describe input-related demands specific to audition including source (aspects of the signal, e.g., accented or rapid speech, frequency content), transmission (aspects of the environment, e.g., noise, reverberation), listener (e.g., sensory and cognitive abilities), message (e.g., vocabulary, semantic context), and context (e.g., situational script) factors that influence arousal and available cognitive capacity. Like Kahneman’s model, FUEL suggests that the amount of cognitive capacity available varies with the listener’s state of arousal. FUEL also posits that additional influences related to task demands and intrinsic motivation will impact the listener’s allocation of attention to the task.

In an alternative limited capacity approach, Lavie [62] attempted to overcome shortcomings of early and late filter theories by incorporating aspects of both models and focusing on the limited capacity of the processing system. In the load theory of attention, Lavie incorporates two primary mechanisms involved in selective attention that can be likened to early and late filters: a passive perceptual selection and an active cognitive control [61]. Lavie posits that the amount of load imposed by a given task upon either of these mechanisms varies the degree to which irrelevant information can be processed within the system. In instances of low perceptual load, both relevant and irrelevant inputs are fully processed somewhat automatically, and the active cognitive control mechanism, or higher order cognitive functions like working memory, acts to identify relevant input and select an appropriate response. Once the capacity of the perceptual system is exceeded, perceptual selection reduces the processing of irrelevant inputs as described by early filter theories. From this, Lavie concludes that attention is only truly selective in instances of high perceptual load as otherwise all inputs are processed automatically until capacity is reached [61]. With high cognitive load, less cognitive capacity remains for the active regulation of irrelevant inputs [62]. Therefore, distractors are more likely to interfere in instances of high cognitive load because fewer cognitive resources are available to focus attention on task-relevant information and appropriate response selection [62].

Overall, behavioral studies suggest that perceptual limitations are more influential in attentional selection than response competition [59,63] which provides greater support for an early over late filter theory. Even “early” filter theories describing selection based on physical characteristics of the signal require some degree of categorization and distinction between the signal and competing noise. However, early perceptual filter limits in the auditory system are difficult to define and evaluate [64]. Much of the work investigating perceptual filter limits relates to vision [61,65,66,67], but more recent studies have attempted to adapt visual paradigms for the auditory modality [68,69,70,71]. Yet, studies applying similar concepts to investigate perceptual load (i.e., varying the number of items presented, the level of similarity between target and non-target items, the number of perceptual operations required by the task) result in mixed outcomes with some revealing attentional load differences while others do not [64].

Ultimately, the selection process in voluntary attention requires a determination of what inputs are most relevant and will become the object of attentional focus [54]. This suggests that some sort of higher order processing is required for the efficient and accurate selection and filtering of relevant from irrelevant inputs. Attentional selection may reinforce early filtering mechanisms via top-down influences that enhance the neural representation of attended speech signals during active speech processing [72]. For instance, in the auditory system, attention may bolster early speech encoding via corticofugal connections between cortical areas and lower brainstem pathways enabling more efficient filtering and attentional focus on the relevant speech stream [34,35,37,38,73,74]. However, where within the auditory system attentional effects may be observed and to what extent attention modulates early auditory encoding during active speech processing remains unclear and cannot be determined from behavioral studies alone.

## 3. Anatomical Studies

Frontal, temporal, and parietal areas, together with heteromodal sensory association regions, all contribute to a distributed network of cortical connections that subserve attention [75]. Attention deficits are largely attributed to dysfunction within the frontostriatal network that is responsible for executive functions related to working memory and cognition and to abnormalities in networks distributed throughout the cortex. The regions that have been primarily implicated in attention deficits are the dorsolateral prefrontal cortex and dorsal anterior cingulate cortex [76,77], inferior parietal lobule [78], corpus striatum [79], cerebellum [77], and myelinated connections that link these structures [80]. Whether attention deficits are the result of a delay in brain maturation or due to a deviation from typical development has been debated, but there is evidence that individuals with attention difficulties demonstrate maturational delays in gray matter thickness and white matter volume into adulthood [81,82]. While research into neuroanatomical differences in individuals with attention weaknesses have focused primarily on cortical and cerebellar regions, there is some evidence of impaired attention and executive functioning in patients with isolated brainstem lesions [83,84]. The brainstem locus coeruleus projects to the prefrontal cortices, and its noradrenergic system has been implicated in problems with response inhibition and working memory in attention deficit patients [85]. The majority of this evidence suggests that attention deficits are due to differences in the structure and/or function of anatomical regions within subcortical and cortical networks known to be essential for attention, but the impact of these abnormalities on different sensory domains is not known. Investigations into whether these structural defects lead to perceptual and comprehension deficits across different sensory modalities is beyond the scope of this review, but a fundamental question is whether comprehension of complex auditory information can be directly attributed to anatomical differences within the attention network or if the delays in development of attention-related anatomy is due to weaknesses in sensory processing through brainstem pathways.

## 4. Electrophysiologic Studies

Efforts in electrophysiology help refine the time course of selective attention in speech processing. Although attentional theories provide an analytical framework and anatomical studies identify key neurological structures involved in attentional processes, the question remains as to where within the auditory system attention begins to influence the encoding and subsequent processing of sensory inputs. Most studies have focused on the role of auditory cortex in selective attention [36,86,87,88,89] as cortical regions are thought to integrate bottom-up physical, acoustic information about the stimulus and top-down influences related to the information’s relevance to a task [48]. However, others have investigated the potential role of attention on earlier processing at the level of the brainstem [36,37,73,90,91,92].

### 4.1. Late Auditory Evoked Potentials

Studies evaluating attentional effects on cortical activation reveal attentional modulation of auditory cortical responses [86]. Late auditory potentials, occurring approximately 50 ms and later following stimulus onset, consist of three primary components (P1, N1, P2). This response is characterized by a tri-phasic waveform with two positive deflections intervened by one negative deflection and is thought to reflect obligatory, automatic processing triggered by the presence of acoustic stimuli [47]. Picton and Hillyard [36] evaluated the effect of attention on evoked potentials across all levels of the auditory neuroaxis. While they found no significant attentional effects on early brainstem or mid-latency responses, they observed enhanced N1 and P2 amplitudes when participants attended to the auditory stimuli to perform a detection task compared to when auditory inputs were ignored. From these results, the authors concluded that early components of the auditory pathway process acoustic stimuli similarly regardless of the listener’s attentional state while the N1-P2 complex may reflect the processes of early perceptual filtering.

Naatanen [88] suggests attentional enhancements of N1 amplitude are driven by an endogenous “processing negativity” that may last longer than the traditional N1 response. The processing negativity is observed in the Nd component, or the difference wave between the evoked responses of attend and not attend conditions [86]. The Nd component may reflect an early filtering process as it can be elicited when inputs vary in physical characteristics [87] consistent with early filter theories [51,52,54] and its onset latency increases with increasing complexity of the task [87] consistent with load theory [61].

Other non-obligatory, event-related potentials have also been used to evaluate aspects of attentional processing including stimulus salience and novelty. These non-obligatory responses are elicited using special paradigms that include frequent, standard and rare, deviant stimuli that differ in acoustic characteristics (e.g., duration, frequency, intensity, interstimulus interval). The mismatch negativity (MMN) is a response occurring around 150–250 ms following a deviant stimulus [93]. It is a negative deflection observed in the difference wave found by subtracting the event-related potentials for the standard and deviant stimuli. MMN amplitude increases with greater salience of deviant tokens (i.e., when more drastic acoustic differences exist between the standard and deviant stimuli) [93]. Thus, the MMN is often associated with auditory discrimination ability. This response is thought to also reflect pre-attentive, sensory memory since its elicitation does not require attention but does necessitate that a representation of the standard stimuli has been stored in auditory memory [93]. While the MMN does not require attention, some studies suggest that the response amplitude is enhanced when attention is directed to auditory inputs [94] and that MMN amplitude is reduced during heighted attentional focus due to increased task demands [71]. Unlike the MMN, the P3 response, a positive peak occurring approximately 250–500 ms following stimulus onset, is only observed in response to tasks requiring attention and behavioral responses [95]. The P3 is elicited by task-relevant stimuli often in target detection tasks, but unattended stimuli may also produce a P3 response if they are novel, unexpected, or highly intrusive reflecting involuntary shifts in attention [86]. As its amplitude varies with confidence in detection and degree of engagement in the task [95,96], P3 is thought to index the processes of stimulus recognition and classification and response preparation [86,96].

In sum, unattended auditory inputs demonstrate less robust responses in early cortical measures than attended signals, and neural representations of ignored signals are notably reduced or absent in later, higher-order cortical responses [72,97]. Similarly, fMRI studies indicate that attention results in more widespread activation across higher-order cortical structures while activity related to unattended signals remains confined primarily to sensory cortices [98]. This finding supports that unattended information is filtered out early in processing reducing the input to, and therefore load on, the channel. The results of these studies suggest that the filtering mechanisms discussed by Broadbent [51], Treisman [52], and Deutsch and Deutsch [53] are observable cortically and that processing from the periphery through the brainstem occurs automatically with very limited top-down influence.

### 4.2. Early Auditory Evoked Potentials

On the other hand, some studies focusing on attentional modulation of brainstem responses suggest that corticofugal projections alter early neural encoding within the ascending auditory pathway and that the relay nuclei within the brainstem may serve as the early filtering mechanism [34,35,37,73,99]. Brainstem responses reflect the initial neural encoding of an acoustic stimulus. Two primary electrophysiologic measures of brainstem encoding are commonly evaluated, the transient response [i.e., auditory brainstem response (ABR)] and the sustained response [i.e., frequency following response (FFR)] [100]. The ABR is observed following the presentation of a transient signal, often a click or tone burst, and results in a series of waves occurring within 10 ms following stimulus presentation. The sustained response, or FFR, is optimally elicited by periodic stimuli such as tones or vowels and reflects the phase-locking ability of neural generators primarily within the rostral brainstem [100,101]. The FFR mimics the spectrotemporal properties of the acoustic stimulus providing a measure of the fidelity and efficiency of early neural encoding.

Attentional effects have been noted for both transient and sustained responses (summarized in Table 2). When investigating the ABR, Lukas [34] found that responses were more robust and occurred earlier when participants attended to auditory tones than when the tones were ignored suggesting that attention enhances neural synchrony and efficiency in early encoding of attended signals. Similarly, Galbraith and colleagues observed enhanced response amplitudes in FFRs when listeners attended tone or vowel stimuli [37,73,74]. Galbraith et al. [73] also found enhanced phase-locking to the attended stimulus fundamental frequency (F0), or the lowest resonant frequency within the stimulus, during a dichotic vowel listening task. The F0 reflects voice pitch encoding which serves as an important cue for segregating a voice of interest from background noise or competing speech [21,100]. These results imply that directed attention enables early selective filtering based on physical characteristics of the stimulus (i.e., pitch) and improves neural encoding of target signals. Overall, the authors of these studies conclude that observed changes with selective attention are due to corticofugal projections acting to suppress irrelevant inputs within the auditory brainstem [34,35,37,73,74]. This indicates a complex interaction between higher order cognitive processes and early sensory encoding.

At the same time, numerous other studies evaluating attentional effects on brainstem responses indicate no attentional modulation of the response and suggest that processing at this level is automatic and pre-attentive (see Table 2). The majority of studies evaluating attentional effects on the transient brainstem response have reported no effects of attention [36,102,103]. This is evident as the transient response is largely unaffected by the state of the listener and can be obtained when the listener is asleep or sedated [100]. Likewise, other studies found no attentional effects when investigating the top-down attentional modulation of sustained responses [90,106]. As previous studies demonstrated enhanced FFRs with attention, Varghese, Bharadwaj and Shinn-Cunningham [90] hypothesized that selectively attending to auditory inputs would enhance the fidelity of phase-locking within the FFR. When responses were compared across conditions varying in attentional demand, no differences in FFR phase-locking were noted while cortical responses demonstrated attentional modulation. As effects of attention were observed only at the cortical level, it was concluded that top-down attentional influences on the brainstem response are minimal, if present at all, during active speech processing.

Ultimately due to contradictory results, attentional influences on brainstem responses, particularly the FFR, remain highly debated and unclearly defined. The seemingly oppositional conclusions in the literature may be attributed to differences in methodology, task demands, signal-to-noise ratio of the electrophysiologic responses, or stimulus properties across studies [90,104]. For instance, Bidelman and Powers [107] suggest that no fewer than 1000 stimulus presentations are required to adequately reduce background EEG noise and obtain an accurate representation of the neural response. However, as few as 500 trials are analyzed in at least one study [74] potentially resulting in inaccurate measures of neural encoding and spurious conclusions.

Acoustic properties of the stimulus may have also influenced previously reported results. A recent study by Holmes, Purcell, Carlyon, Gockel and Johnsrude [104] revealed that attentional effects are observed for responses to stimuli with low (i.e., <110 Hz) but not high frequency modulation rates. The authors attributed the differing attentional effects on the FFR to cortical contributions to the recorded “brainstem” response for stimuli containing spectral content below cortical phase-locking limits [104]. While subcortical sources are the primary generators of the FFR, the scalp recorded response reflects the summation of neural activity from multiple generators; therefore, responses to stimuli containing frequencies below the phase-locking limit of auditory cortex may also include cortical phase-locked activity [101,105,108]. In these instances, the cortical contributions may not be influencing the brainstem via corticofugal projections per se but rather may produce observable attentional enhancements in the FFR response pattern.

Despite the insight gained from the previously discussed studies, it remains unclear whether attention modulates neural encoding at early levels of processing and whether corticofugal projections contribute to active speech processing in difficult listening environments. It is possible that attention does influence the initial encoding of acoustic stimuli, but previously used measures were not sensitive enough to consistently detect efferent modulation at such an early level of auditory encoding. Further study is necessary to elucidate these contradictory results.

### 4.3. Simultaneous Recording of Brainstem and Cortical Evoked Responses

One such method that may assist in clarification is the simultaneous recording of brainstem and cortical responses. This recording technique enables the evaluation of responses within each level of the auditory neuroaxis; thus, providing a more complete representation of processing throughout the pathway [109,110]. In these recordings, FFRs provide measures of the precision of initial neural encoding of spectrotemporal properties of the stimulus while cortical responses provide insight related to later sensory and perceptual processes [110,111]. Simultaneous recording also affords the evaluation of the interaction and neural transmission between levels of the auditory pathway related to bottom-up and top-down processing [112,113,114]. Importantly, simultaneous measures can evaluate online speech processing of the same stimuli for brainstem and cortical responses which accounts for potential confounding variables of differing stimulus characteristics and participant state across separate recording sessions.

Simultaneous recordings have been used previously to investigate the hierarchical auditory processing of non-speech and speech sounds in the context of pitch salience [113], concurrent sound segregation [115], auditory plasticity [116,117], categorical perception [112,116,118], speech-in-noise perception [114,119,120,121], aging [118,120,121], and hearing loss [114,121]. These studies reveal that the fidelity and efficiency of early encoding of auditory inputs in lower brainstem regions influence processing at higher auditory cortical areas [113,119,122]. Interestingly, Krishnan and colleagues [113] report that behavioral performance was better predicted when both brainstem and cortical responses were considered together than each separately. Furthermore, recent studies reveal that differences in neural transmission strength between auditory brainstem and cortex contribute to differences in SIN perception irrespective of hearing status [29,114] and may be used to predict SIN success [114,119].

Therefore, simultaneous recordings may more accurately and comprehensively capture the complexities of the dynamic auditory system. Future studies may use similar recording techniques to investigate the influence of higher order cognitive processes, such as attention, on earlier auditory processing centers. Top-down attentional influences may function to enhance, or fine-tune, early processing strengthening the representation of the signal throughout the ascending auditory pathway. In fact, Marsh and Campbell [50] recently proposed a new early filter model expanding on Broadbent’s early filter theory [51]. Marsh and Campbell [50] posit the brainstem serves as the early filtering mechanism for expected sounds based on prior context. Top-down modulation serves to selectively enhance target signals while suppressing unattended, competing noise. Using simultaneous recording techniques to evaluate the role of attention when processing speech in adverse listening conditions will provide evidence to support or refute proposed models of attention as well as provide novel insight into afferent and efferent interactions and attentional influence within the auditory pathway. Thus, simultaneous recording may more thoroughly characterize the influence of attention on auditory processing during online speech perception.

## 5. Conclusions

Attentional theories and models provide theoretical frameworks from which to base the empirical study of neural mechanisms of attention during speech processing. As selective attention is required to effectively communicate in difficult listening environments, it is vital to obtain a more thorough understanding of how ascending levels within the auditory system interact with higher order cognitive processes to facilitate perception and comprehension of speech material. A better understanding of neurophysiological contributions to attentional processes from subcortical and cortical networks will help characterize its operations in typical systems, identify potential areas of weakness in disordered systems, and ultimately inform and optimize intervention techniques to facilitate speech understanding.

Together with evidence from studies investigating the effects of attention on early and late evoked potentials as reviewed here, novel methods integrating brainstem and cortical responses through simultaneous recordings have the potential to supplement important information about contributions from brainstem pathways in individuals with speech comprehension difficulties. A significant contribution could be objective evidence of differences in early sensory encoding within the brainstem, early perceptual processing within auditory cortex, and/or neural transmission between the subcortical and cortical regions of the auditory pathway as discussed by Humes [16] and Moore [15]. Research evidence that illustrates the role of attention throughout interactive mechanisms in the ascending and descending pathways of the auditory system, detailing how a processing deficit in one area can contribute to weakness in another, is needed to disentangle the underlying operations contributing to impaired perceptual processing and/or more global attentional deficits.

Clarifying the role of attention within complex interactions that likely underlie common communication pathologies is particularly useful for disorders that present with diverse and varied symptoms across individuals such as auditory processing disorder (APD). As defined by the American Speech-Language-Hearing Association, APD is “a deficit in neural processing of auditory stimuli that is not due to higher order language, cognitive, or related factors” [123]. Individuals with APD commonly demonstrate difficulties with SIN, problems attending to complex verbal information, deficits in identifying competing information as in dichotic listening tasks, and problems following multi-step, auditorily presented instructions. Some researchers deny sensory contributions to APD and attribute these processing problems primarily to cognitive factors [124], but evidence of perceptual effects following periods of sensory deprivation presumed to alter processes within the auditory brainstem suggest that cognitive effects may be secondary in some patients with comprehension difficulties [125,126]. Individuals with suspected APD often demonstrate dichotic listening deficits that are also attributed to impairments in general attention or in the cognitive control necessary to appropriately direct attention during challenging listening tasks [127,128,129]. In the classic chicken versus egg dilemma, researchers have yet to characterize whether innate or acquired differences in auditory nuclei within the brainstem, midbrain, and/or thalamus lead to maturational delays and functional alterations within cortical networks that subserve attention or if abnormalities within the frontostriatal network alone alter how complex auditory signals are ultimately processed in the cortex. In parallel with ongoing research regarding the negative effects of neurological damage in the cortex on an individual’s attentional capacity, investigations are needed into how abnormalities within lower level structures of the auditory brain may also interfere with normal processing of complex auditory information such as listening in background noise and with competition. Investigations are needed into the critical links between complex sensory processing in the brainstem and effective ascending transmission of neural signals into and through cortical neural networks that subserve attention, working memory, and cognition. A greater understanding of how functional deficits may arise from innate or developmental alterations within bottom-up sensory processing pathways can lead to improvements in clinical assessments and interventions. Objective, electrophysiological measures that incorporate neurophysiology with attention and characterize functional connections between lower- and higher-level processing centers can aid in these investigations. Without the confounds of language and working memory that plague behavioral test protocols and with the ability to manipulate attention as an independent variable, simultaneous recordings of activity within brainstem and cortical regions may aid in separating global attentional deficits from auditory-specific deficits in patients who struggle when listening in background noise. Future studies should aim to disentangle the sensory vs. perceptual factors contributing to speech-in-noise deficits and identify neural changes that predict the presence and severity of such difficulties. At that time, protocols may be adapted making them more practical and accessible for clinical use. FFRs and ERPs can be collected clinically using systems commonly used for ABR diagnostic testing. Indeed, some current systems include capabilities for eliciting ABRs to speech stimuli (e.g., /da/). Similar paradigms could easily be employed to assess deficits in speech processing.

Assessments that identify unique weaknesses in specific auditory processing skills have greater potential to lead toward targeted interventions. In some areas of auditory clinical practice, individualized and client-centered treatments, together with appropriate counseling strategies, have resulted in greater adherence to recommended interventions, improved functional status, and enhanced patient satisfaction [130,131]. Clinical procedures that can isolate a patient’s functional deficits and integrate them with personal and environmental factors are more likely to improve rehabilitative outcomes and increase satisfaction. Despite the availability of an extensive battery of behavioral and objective assessments, many clinicians struggle to specify the processes that are most problematic for individuals across the lifespan with SIN deficits. An individual’s ability to effectively communicate in a world of complex auditory signals depends on a continuous integration of active processes within both bottom-up and top-down neural pathways. The primary goal of clinical intervention should be to address and remediate deficits within an integrated approach that considers attention, working memory, cognition, and compensatory strategies. While that goal remains elusive in auditory rehabilitative care, new objective measurements of how attention intersects with speech processing tasks throughout the auditory brain can assist in the development of integrated interventions for persons with SIN difficulties. Strategies that delineate the role of global factors such as attention in common processing disorders are consistent with the movement toward individualized medicine and patient-centered care as supported by the World Health Organization and allied health professionals.

## Figures and Tables

**Table 2:** Summary of studies evaluating attentional influence on evoked brainstem responses.

Response	Attentional Effects?	Study	Results
Transient (ABR)	Yes	Lukas [34,35]	Enhanced amplitude and decreased latencies when attending to auditory stimuli
	No	Picton, Hillyard, Galambos, & Schiff [102]	Enhanced cortical response but no attentional change observed in ABR
	No	Picton & Hillyard [36]	No significant attention-related changes in auditory evoked response until cortical N1-P2 ERP components
	No	Woods & Hillyard [103]	No change in ABR amplitude or latency as a function of attention
Sustained (FFR)	Yes	Forte et al. [92]	More robust FFR reported when attending than when ignoring speech
	Yes	Galbraith & Doan [74]	Enhanced FFR amplitudes with attention
	Yes	Galbraith et al. [73]	Enhanced FFR F0 for attended stimuli
	Yes	Galbraith et al. [37]	Attentional enhancements of overall FFR amplitude
	Mixed	Holmes et al. [104]	Effects of attention observed for low- (<110 Hz) but not high-frequency stimuli
	Mixed	Hartmann & Weisz [105]	Attentional modulation only within cortical generators of FFR—not brainstem
	No	Galbraith & Kane [106]	Enhancement of cortical ERPs but stable FFRs with attention
	No	Varghese et al. [90]	Attention-related enhancement noted in cortical responses but not FFR

## Data Availability

Data sharing not applicable.
